# Supplementary figures and images for: A Disintegrin and Metalloprotease 12 Promotes Tumor Progression by Inhibiting Apoptosis in Human Colorectal Cancer
Source: Cancers (Basel). 2021 Apr 16;13(8):1927. doi: 10.3390/cancers13081927 (PMC8073784; doi:10.3390/cancers13081927)

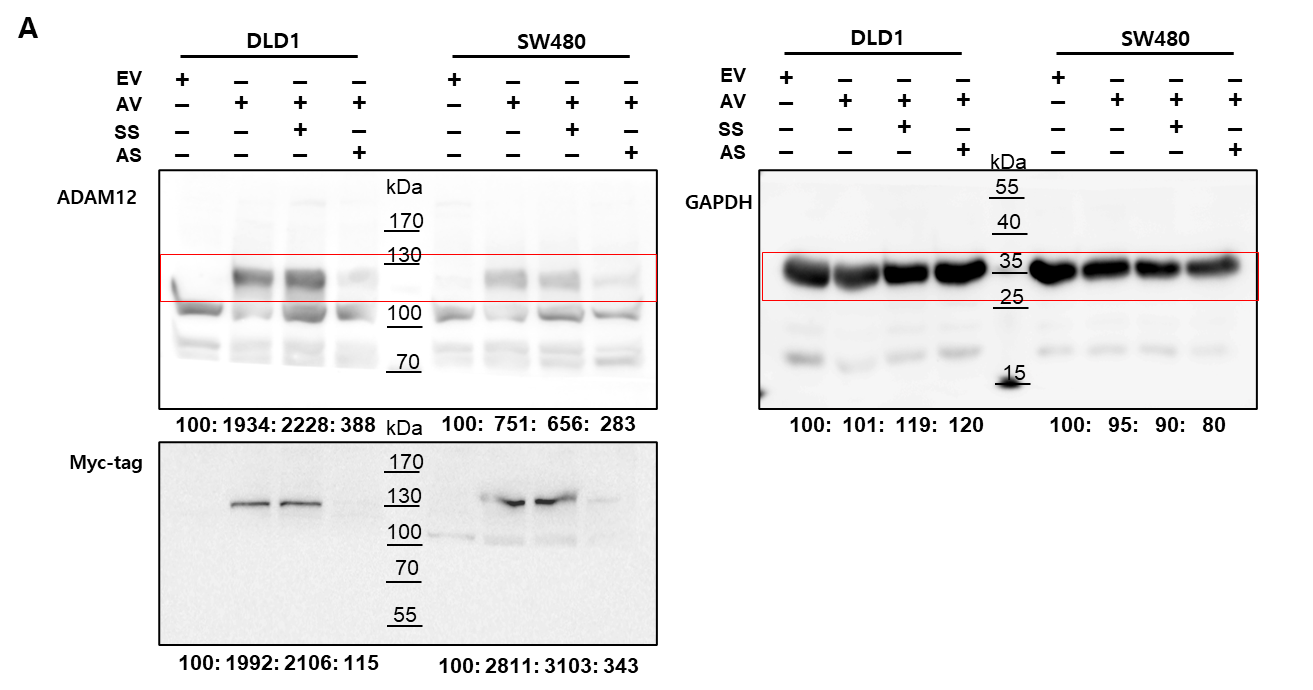

Supplement: Supplementary file 1 [file cancers-13-01927-s001.zip › cancers-1143044-supplementary/Figure S1 supplementary data(revised).tif]

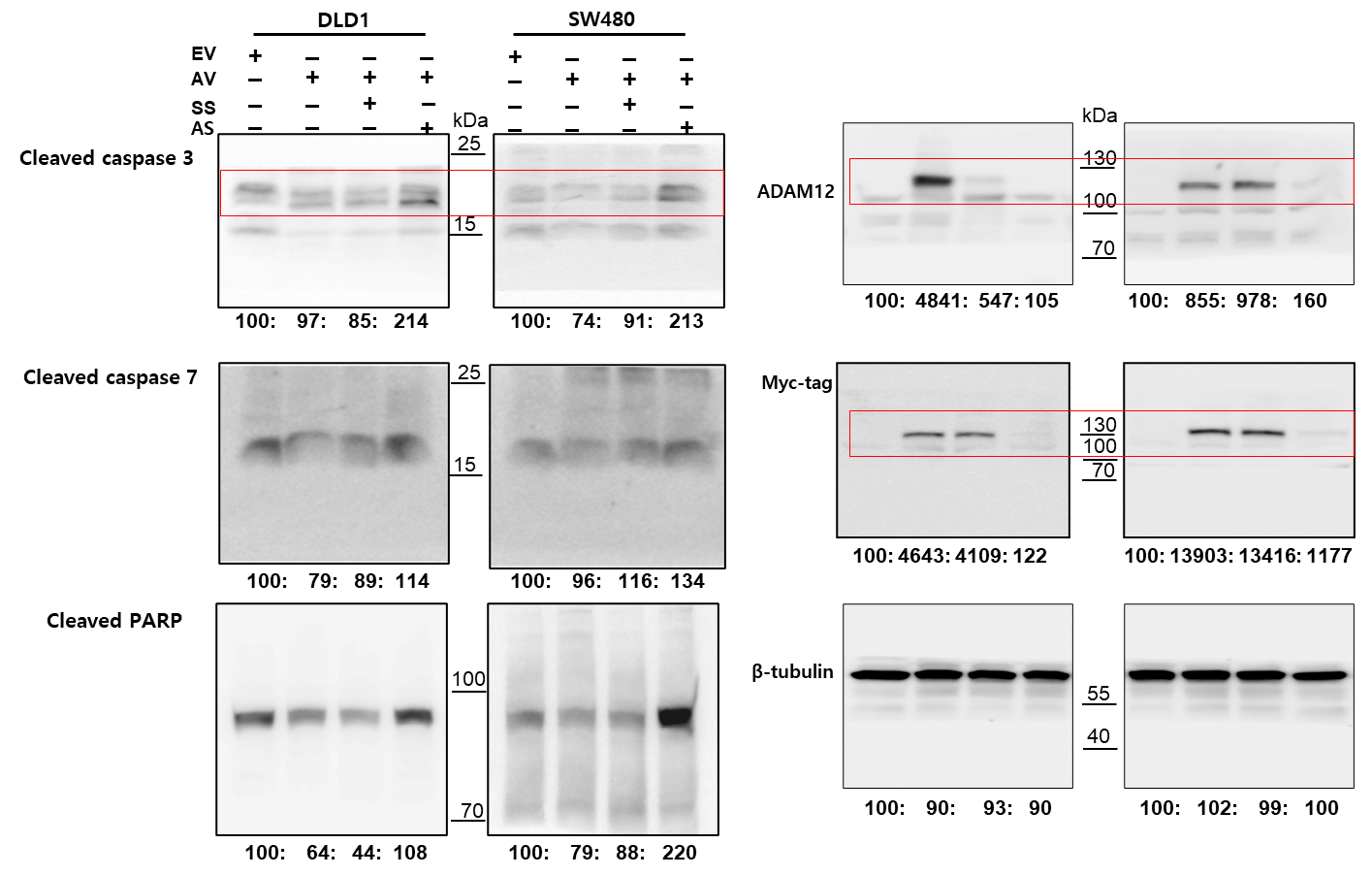

Supplement: Supplementary file 1 [file cancers-13-01927-s001.zip › cancers-1143044-supplementary/Figure S2 supplementary data(revised).tif]

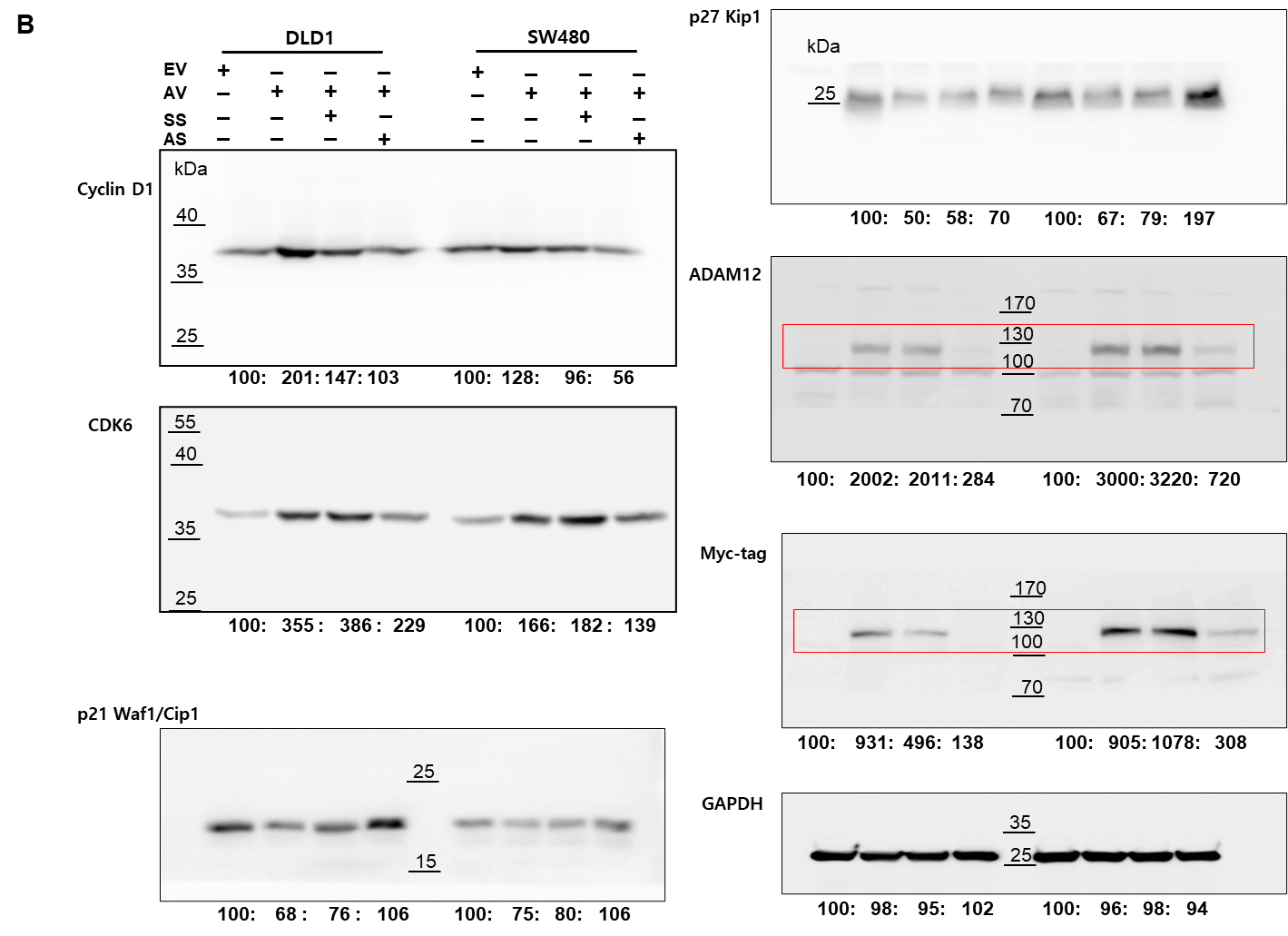

Supplement: Supplementary file 1 [file cancers-13-01927-s001.zip › cancers-1143044-supplementary/Figure S3 supplementary data(revised).tif]

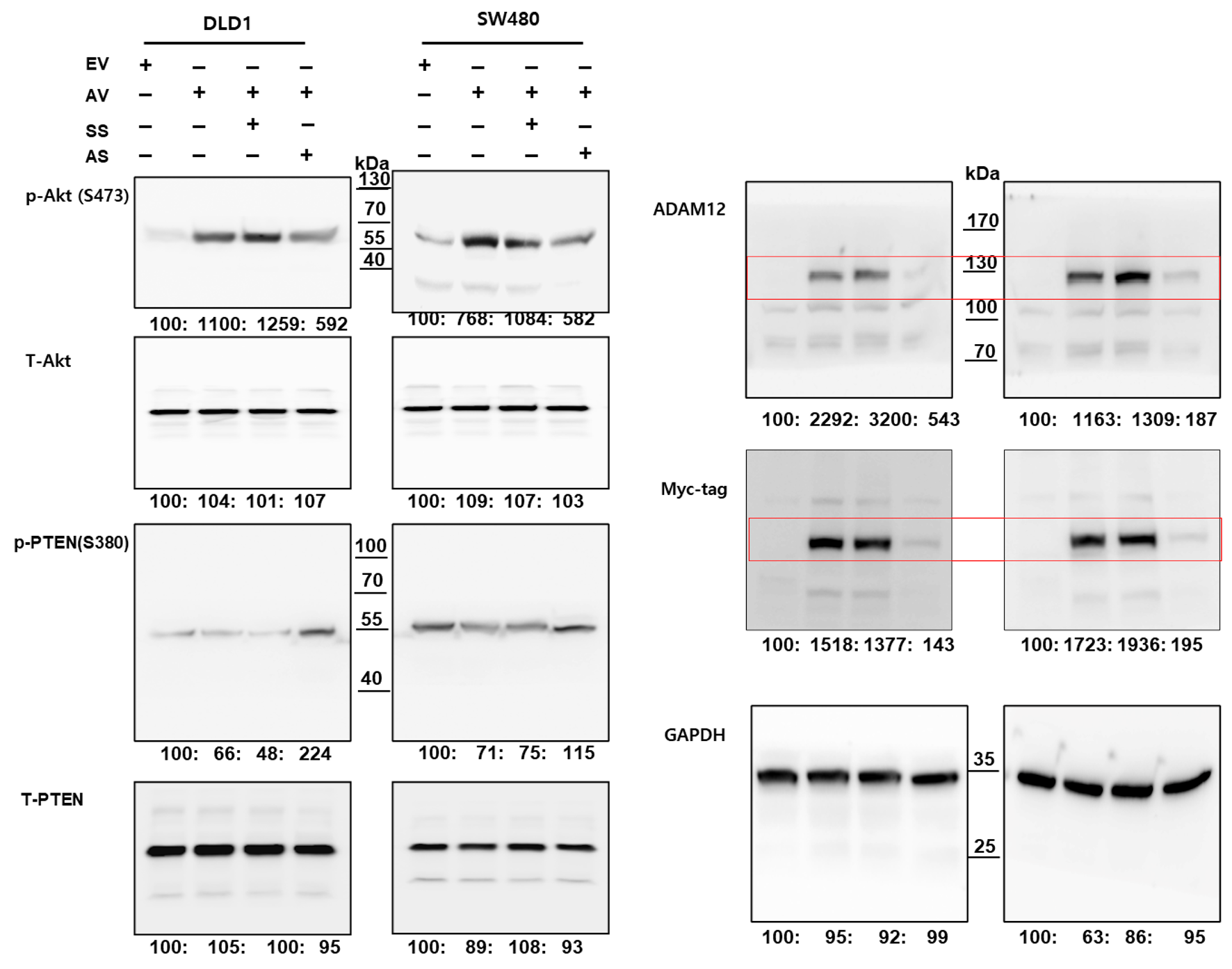

Supplement: Supplementary file 1 [file cancers-13-01927-s001.zip › cancers-1143044-supplementary/Figure S4.tif]
